# Neuropsychological Aspects of Children’s Somatic Disorders in Chronic Diseases: Diabetes and Short Stature in the Developmental Period

**DOI:** 10.3390/biomedicines11113089

**Published:** 2023-11-17

**Authors:** Maia Stanisławska-Kubiak, Katarzyna Wiecheć, Katarzyna Anna Majewska, Grażyna Teusz, Ewa Mojs, Andrzej Kędzia

**Affiliations:** 1 Department of Clinical Psychology, Poznań University of Medical Sciences, 61-701 Poznan, Poland; k.wiechec@interia.eu (K.W.); ewamojs@ump.edu.pl (E.M.); 2 Center for Trauma, Crisis Add Growth, SWPS University of Social Sciences and Humanities, 61-701 Poznan, Poland; 3 Department of Pediatric Diabetes, Auxology and Obesity, Poznań University of Medical Sciences, 60-572 Poznan, Polandakedzia@ump.edu.pl (A.K.); 4 Faculty of Educational Studies, Adam Mickiewicz University, 60-568 Poznan, Poland; jasmin8@amu.edu.pl

**Keywords:** cognitive processes, chronic diseases, children, type 1 diabetes, isolated growth hormone deficiency

## Abstract

Intellectual functioning studies carried out amongst children indicate that chronic diseases like type 1 diabetes and growth hormone deficiency (GHD), may, but do not necessarily, result in intellectual loss. Cognitive functions may decline as a child becomes older, as a disease persists over time and/or due to non-compliance with treatment recommendations or high stress levels. This study aimed to assess the cognitive functioning of children and youths with T1D and GHD-related short stature compared to healthy children. Methods: The study was carried out on 88 children with type 1 diabetes, 38 children suffering from short stature caused by (GHD), as well as a control group comprising 40 healthy children. Weschler’s tests were applied to measure intellectual and cognitive functions. Results: The results suggest that for children suffering from type 1 diabetes and short stature, their chronic childhood diseases per se do not impair cognitive development. It was observed that the higher the age of chronically ill children and the longer the disease persists, the lower their scores in individual cognitive subtests. For healthy children, age is correlated with the acquisition of particular skills and higher scores in specific subtests. Conclusions: On the basis of qualitative analysis of the cognitive functions subject to the study and close clinical observation of chronically ill children, we have been able to conclude that chronic diseases may alter cognitive functioning.

## 1. Introduction

A chronic disease in childhood affects various body functions. These also includes the human cognitive processes. The quality of life of a child suffering from a chronic disease is greatly affected by the intensity of symptoms and the duration of a particular health problem. Conditions that lead to changes in, for example, the diet (diabetes) or appearance, such as very short stature, are very debilitating. Consequences of a disease may involve negative peer group interactions, increased focus on one’s own body and may lead to lower self-esteem and withdrawal from social interactions or other psychological difficulties such as depression or eating disorders. Furthermore, sufferers may become reluctant to work or study, their motivation and self-control may decrease, and they may become less responsible. A child may develop different coping mechanisms. How they (and their loved ones) will actually cope with a disease depends on both internal resources such as personality, temperament, intelligence level, stress coping strategies, emotions, behaviour when confronted with the disease, and external resources which include support from carers, doctors, nurses and psychologists [1]. A neuropsychological assessment provides information about the level of functioning of a subject in relation to expected developmental norms. It also addresses intrapsychic differences in the development of specific cognitive functions and emotions as well as social functioning. It is difficult to pinpoint the nature of these changes in children and youths; this is due to the fact that the first symptoms of a disease occur in children at different psychosocial development stages; different children react to and experience their disease as well as the treatment process in very individual ways; they have different resources at their disposal at the time of diagnosis and they also receive varying degrees of support from those closest to them. On top of that, there are two theories with respect to the development and functioning of the nervous system in children with a chronic disease. According to the first, as the nervous system is immature, it is particularly vulnerable during childhood. According to the second theory, the plasticity mechanism triggers compensation, and the affected functions are taken over by other brain areas [2].

A direct comparison of youth with short stature to those with type 1 diabetes would be a significant addition to existing research. The study on short-statured and type 1 diabetes children’s psychological adjustment has depended on comparisons with normative, healthy samples. Therefore, rather than being tied explicitly to disease, previous findings may indicate behavioural and emotional issues caused by having a medical condition [3,4,5]. Research also shows that children and adolescents with short stature and children with type 1 diabetes achieve similar results in terms of quality of life. Both disorders are related to maintaining an appropriate diet (in the case of short stature treated with rhGH, there is a risk of type 2 diabetes) and persistent treatment associated with injecting the drug using a special pen [6,7]. One way to reduce any adverse effects of physical disease on psychological functioning would be to contrast children with GHD with a group of different endocrine disorders like diabetes (also including a control group of healthy children) [8].

In this article, we take a close look at children with type 1 diabetes and short stature resulting from GHD in the context of cognitive functioning and its role in therapeutic decision-making.

Type 1 diabetes (t1D) is one of the most common chronic diseases of the development period. The recommended treatment entails a functional, intensive insulin therapy, monitoring blood glucose levels, calculating insulin requirements, administering insulin and/or glucose as needed, controlled nutrition, exercise as well as use of medical equipment such as pens or an insulin pump [9]. Doctors have to take into account specific aspects associated with caring for and dealing with children and youths with t1D. These include changes in insulin sensitivity related to physical development and maturing, [10], neurological vulnerability to hypoglycaemia and hyperglycaemia in young children and the possible adverse neurocognitive effects of diabetic ketoacidosis [11,12] as well as attention to family dynamics [13] to optimise the therapeutic process.

Short stature is defined as a body height below the third percentile (2 standard deviations from the mean), as assessed on centile charts appropriate for age, gender and population [14]. Short stature may be attributed to a number of factors. Including acquired diseases or congenital disorders [15]. Growth disorders are usually multifactorial in nature. Therefore, growth deficiency diagnosis is a complex process, requiring a lot of information regarding the patient’s condition and history [16]. Growth hormone deficiency is associated with insufficient secretion of growth hormone and can occur as an isolated condition. For the treatment of this disorder, recombinant human growth hormone (rhGH) is used, administered every evening by subcutaneous injections. The course of treatment is usually monitored during outpatient visits or possibly short-term hospitalisation, during which the effectiveness of treatment (growth rate evaluation) and possible adverse effects of rhGH therapy are assessed [17].

The aim of the study was to assess the cognitive functioning of children and youths with t1D, and GHD-related short stature in comparison with a group of healthy children.

## 2. Materials and Methods

The study was carried out on a group of 88 children with t1D, and 38 children with GHD-related short stature.

During the course of the study, all patients were periodically admitted to the Karol Jonscher Clinical Hospital of the Poznań University of Medical Sciences as part of routine check-ups established for these patients.

All children with GHD-related short stature were included in this study prior to rhGH treatment.

All children with t1D were long-term-treated with subcutaneous insulin, and presented no acute nor chronic complications of the disease at the time of examination.

A control group comprising 40 healthy children was based on a population from the Greater Poland region, found using the snowball sampling method. The inclusion criteria were residence in Poznań, age distribution similar to the studied groups of children with t1D and GHD, lack of chronic diseases, and intelligence within the norm for the population (Table 1). Additionally, the group of healthy children does not differ in terms of parents’ education and the children’s financial status.

A neuropsychological examination assessing abilities in selected cognitive processes, performed with the Wechsler test for children was the primary research method.

The Wechsler Intelligence Scale for Children (WISC-R) was administered. This test entails verbal and non-verbal scales and therefore engages a variety of intellectual functions, thus enabling a comprehensive diagnosis of intellectual ability. The scale is made up of 10 core tests (Information, Picture Completion, Similarities, Matrix Reasoning, Arithmetic, Block Design, Vocabulary, Visual Puzzles, Comprehension, and Coding) and 2 alternate tests (Digit Span, Labyrinths). Half of the test is verbal, and half is non-verbal. The two types of tests are arranged in alternating order [18]. The WISC-R scale is based on raw and scaled scores. Raw scores are obtained by adding the scores obtained in a given subtest. The way in which answers are scored and evaluated is strictly defined by specific instructions, different and specific to each subtest. Subtest results can only be interpreted after raw results have been scaled. Scaled scores are a standard score scale based on a normal distribution for a given population [18]. The sum of all 10 scaled subtests for verbal and non-verbal scales determines the overall Intelligence Quotient (IQ). Appropriate tables included in the test manual, which use the place of the tested child against a representative population as a quantitative indicator, facilitate converting scaled scores into IQ. Test tasks require the use of both verbal and performance strategies.

On average, it took approximately 1.5 h to test a person. After the test, feedback was provided to each patient or their carers on cognitive test results.

SPSS Statistics 28 for the Wechsler Intelligence Scale for Children (WISC-R) scores was used to obtain descriptive statistics and verification of the conformity of the variable distributions to the normal distribution. The research was approved by the local Ethics Committee at Poznan University of Medical Sciences. Parents or legal guardians of all included children gave their informed consent.

Age, IQ scores and IQ subscales of diabetes, short stature, and control groups were compared with the Kruskal–Wallis H and Dunn’s tests with the Bonferroni correction. The effect size was tested using the Epsilon square coefficient (ε2R). Spearman’s rho correlation coefficients analysed age according to a type of chronic disease and intelligence. All hypotheses were bidirectional, and the statistical significance level was considered as *p* < 0.05.

## 3. Results

Results of the Kolmogorov–Smirnov test presented in Table 2 show Overall IQ, Verbal IQ and Non-Verbal IQ variables to be normally distributed. Information, Similarities, Arithmetic, Vocabulary, Comprehension, Digit Span, Picture Completion, Matrix Reasoning, Block Design, Visual Puzzles and Coding do not follow a normal distribution.

As the majority of variables are not normally distributed, non-parametric tests will be used to verify the hypotheses.

Kruskal–Wallis H test results presented in Table 3, show that there are significant differences for the following variables according to the type of disease: Information (H = 10.38, *p* = 0.006), Comprehension (H = 5.81, *p* = 0.050), and Coding (H = 9.01, *p* = 0.011).

The results of Dunn’s post hoc test showed the following:

For Information, children with chronic type 1 diabetes have lower scores than children with short stature and healthy children.

For Comprehension, children with chronic type 1 diabetes and children suffering from short stature have higher scores than healthy children.

For Block Design, children with chronic type 1 diabetes and healthy children have lower scores than children with short stature.

In addition, an analysis effect size, tested using the Epsilon square coefficient (ε2R), showed a weak effect of the chronic-disease-type independent variable on the following dependent variables: Information (ε2R = 0.07), Comprehension (ε2R = 0.04), and Block Design (ε2R = 0.05). In addition, an analysis effect size, tested using the Epsilon square coefficient (ε2R), showed a weak effect of the chronic disease type independent variable on the following dependent variables: Information (ε2R = 0.07), Comprehension (ε2R = 0.04), and Block Design (ε2R = 0.05).

The relationship between the children’s age and their level of intelligence was analysed separately in groups according to the type of chronic illness.

Spearman’s rho (rs) test results, presented in Table 4, show the following:

The age of children with chronic type 1 diabetes shows a significant weakly negative correlation with Verbal IQ, Similarities (rs = −0.276) and Vocabulary (rs = −0.365), and a significant, weak positive correlation with Digit Span (rs = 0.312). This means that for children with diabetes, the older they are, the lower their scores for the Verbal IQ, Similarities and Vocabulary scales and higher for the Digit Span scale.

The age of children with short stature shows a significant weak negative correlation with Similarities (rs = −0.282) and Vocabulary (rs = −0.342) and a significant, weak positive correlation with Visual Puzzles (rs = 0.320).

This means that for children with short stature, the older they are, the lower their scores for the Similarities and Vocabulary scales and higher for the Visual Puzzles scale.

The age of healthy children shows a significant, moderate positive correlation with Visual Puzzles.

This means that for healthy children, the older they are, the higher their scores for the Visual Puzzles scale (rs = 0.448).

## 4. Discussion

The impact cognitive functions have on somatic and emotional health as well as its importance have been indicated by numerous studies. This article may be categorized as one which analyses cognitive difficulties in the context of a chronic disease during the development period spanning childhood and adolescence. Cognitive functions oversee, control and guide an individual’s actions. As higher mental actions, they are associated with abilities such as goal setting tasking into account long-term consequences, initiation of purposeful actions, creation of various possible alternative reactions, abstract logical thinking, or modification of one’s own activity in relation to changing conditions. Executive functions are involved in virtually every human activity, perhaps with the exception instinctive reactions or the most automated and learned actions [19,20]. Different studies on t1D suggest that even high levels of general intelligence do not protect from difficulties with verbalization and problem-naming, predicting and planning behaviour, and piecing together information and from difficulties in applying acquired knowledge (e.g., solving new problems). Recent studies show that the alterations associated with t1D affect the white matter’s microstructural integrity in the thalamus, hippocampal, and parietal lobe regions [21]. The general cognitive functioning decrease with increasing HbA1c value [21]. Neurocognitive deficiencies, including slower processing speed, less attention, and poorer executive skills, are thought to be caused by these alterations [22]. Patients with t1D whose average glycated haemoglobin (HbA1c) concentration was less than 7.4% performed significantly better on tests measuring the rate of thought processes and visual–motor coordination than people whose average HbA1c was greater than 8.8% [23]. These challenges may harm one’s ability to react in challenging situations (related to hypo- and hyperglycaemia), in situations of prolonged emotional tension (related to glycaemic imbalance), or when dealing with challenges related to managing the disease. Research on GDH and rhGH also indicates that long-term treatment with GH and IGF1 considerably improves learning and memory in aged rats with impaired GH production [24]. Additionally, studies show that modifications to the GH/IGF-I axis have a major impact on modifications to various cognitive functions, including memory and executive abilities [25]. Reductions in GH secretion are linked to alterations in cognition (including the emergence of illnesses) and physiology. These effects vanish when rhGH treatment is used. Maintaining proper GH concentration contributes to the optimal course of cognitive processes. The deficit of this hormone leads to a decrease in the volume and integrity of the white matter, hippocampus, thalamus, basal ganglia, corticospinal tract, and corpus callosum. The white matter changes observed concern brain structures that influence the development of cognitive and motor functions (Webb).

Information obtained on the basis of neuropsychological tests also make it possible to monitor a young patient’s functioning from a certain perspective—showing both the patient’s therapeutic progress and cognitive deterioration. Neuropsychological diagnosis in medical practice is a complementary method to better understand a patient’s cognitive and emotional functioning, and to check whether cognitive impairment is due to pathological changes [26]. Studies of children with chronic diseases often highlight that intelligence may be of importance for the course and treatment of the disorder. Individuals with a high intelligence quotient and efficient cognitive functions are more consistent and meticulous when it comes to taking medicines and living longer [27]. Such individuals exhibit significantly better organisation abilities, have better memory, and an increased need for controlling the disease. These components drive individuals to take action aimed at getting better [28]. The process of treating type 1 diabetes requires a range of skills. These include calculating insulin requirements, operating insulin pumps, and analysing blood glucose. On the other hand, treating GHD-related short stature requires a great deal of consistency, rhGH therapy being a long-term procedure, as well as responsibility in terms of following one’s doctor’s recommendations (in terms of administering medicines and consuming food). In this context, an adequate intelligence quotient would be a positive factor in the treatment process. Information about potential cognitive decline as a result in type 1 diabetes, low/high blood glucose, or insufficient growth in GHD patients would be important in terms of identifying the need for potential support and control of the child within the scope of meeting treatment goals.

Interestingly scores decreased for two subtests, Similarities and Vocabulary, across all the chronic diseases studied. Similarities test a child’s ability to think in an abstract manner, identify objects and concepts and the ability to classify (generalise, abstract), think creatively, their creativity, independent thinking skills and plasticity, learning and adaptability. On the other hand, Vocabulary examines skills such as concept formation, the stock of existing knowledge, language skills—defining, verbalising and the ability to learn [18].

Research [29] highlights that even if children with diabetes exhibit moderate or mild cognitive dysfunction, this can affect their daily activities and cause particular difficulties in specific situations.

For example, children and youths with t1D are more likely than their peers without diabetes to perform slightly worse when it comes to tasks requiring sustained attention, rapid processing, memory and visuospatial functioning [18]. Meta-analyses show that executive functions are particularly affected, including processes such as working memory, attention and response inhibition [30].

Executive functions are also studied in children with short stature. Such research show that even if chronically ill children exhibit moderate or mild cognitive dysfunction, this can affect their daily activities and cause particular difficulties in specific situations. Meta-analyses of cognitive processes in chronically ill children show that executive functions are particularly affected, including processes such as working memory, attention and response inhibition [31].

Our study also has some limitations, like the small group of children with short stature compared to those with diabetes. Isolated GHD is an infrequent disorder, and collecting a group of children who met the inclusion criteria (i.e., without comorbidities) was very difficult and took three years. Due to the risk of expiration of the collected measurements, we decided to end the study at the stage of 38 participants. Another limitation may be that the study was conducted based on the Wechsler Intelligence Scale for Children—Modified Version with the manual in the 2nd edition of 2008. The standards of this scale edition have yet to be changed and may, therefore, be outdated. Due to the phenomenon known as the “Flynn effect,” the results (expressed in IQ) obtained by currently tested children are overestimated to those that these children would receive if the norms were current [31,32,33]. For this reason, the control group of healthy children was significant in our study. The third limitation of the study is the gender distribution in the group of children with short stature. It is typical for this clinical group (mainly boys are diagnosed GHD), but at the same time, this distribution is not typical for the entire population. In the field of intelligence research, spatial abilities are the only ones that are systematically revealed gender differences in research. For this reason, we did not emphasize higher results in spatial skills in the conclusions and discussions—because it may be an error resulting from the more significant share of boys in the group of children with GHD.

Slight differences between healthy and children with disease may indicate the appropriate control group selection. Studies on children with t1D and GHD do not indicate significant cognitive differences between children suffering from these diseases and healthy ones. Significant differences in the results would instead indicate an incorrectly selected control group (e.g., by selecting children with too high IQ compared to children with t1D and GHD). At the same time, knowing that the control group may constitute an appropriate reference point—results regarding age and duration of the disease, and decreased results in terms of cognitive functions in GHD and t1D children. No changes or improvement in healthy children may indicate the disease as an essential factor disturbing cognitive processes.

## 5. Conclusions

Neuropsychological analysis of young patients with these chronic diseases is essential information in diagnosing changes occurring during the disease while facilitating the choice of appropriate therapy.

On the basis of qualitative analysis of the cognitive functions subject to the study and close clinical observation of chronically ill children, we have been able to conclude that a disease may alter cognitive functioning. Identifying certain characteristic features of this altered functioning in children and adolescents with chronic diseases is also possible.

For chronically ill children, the older they are, the lower their scores in individual subtests. For children with type 1 diabetes and children with short stature, the results showed lower scores in Similarities and Vocabulary on the Verbal IQ scale.

Age is correlated with better scores in the Visual Puzzles subtest for children with short stature and for healthy children.

For healthy children, age is correlated with no difficulties within the scope of cognitive parameters and even with the acquisition of particular skills and higher scores in specific subtests.

Understanding the patient in terms of their illness is also an important aspect. Early outcome-based interventions help to shape particular skills in children, especially when they are overwhelmed by stress associated with these type of chronic diseases.

## Figures and Tables

**Table 1:** Sociodemographic characteristics group.

	Diabetes (N = 88)		Short Stature (N = 38)		Healthy (N = 40)	
	n	%	n	%	n	%
Female	46	52.3	8	21.1	17	42.5
Male	42	47.7	30	78.9	23	56.5
Age/years						
6	1	1.1	2	5.3	1	2.5
7	7	8.0	5	13.2	3	7.5
8	7	8.0	3	7.9	4	10.0
9	9	10.2	5	13.2	7	17.5
10	6	6.8	4	10.5	7	17.5
11	7	8.0	7	18.4	1	2.5
12	10	11.4	2	5.3	3	7.5
13	11	12.5	1	2.6	6	15.0
14	10	11.4	4	10.5	4	10.0
15	14	15.9	3	7.9	2	5.0
16	6	6.8	2	5.3	2	5.0

**Table 2:** Descriptive statistics for the studied variables.

Variable	Min	Max	M	SD	Kolmogorov–Smirnov	
					Z	*p*
Overall IQ	87	162	114.51	13.08	0.07 *	0.050
Verbal IQ	81	142	111.62	13.28	0.05	0.200
Non-Verbal IQ	69	151	114.19	13.71	0.04	0.200
Information	4	19	10.90	3.04	0.09 **	0.004
Similarities	5	19	12.67	2.71	0.10 **	<0.001
Arithmetic	3	17	11.42	2.96	0.10 **	<0.001
Vocabulary	3	17	11.32	2.64	0.10 **	<0.001
Comprehension	5	18	12.54	2.73	0.11 **	<0.001
Digit Span	2	18	10.55	2.81	0.11 **	<0.001
Picture Completion	4	17	10.42	2.48	0.10 **	<0.001
Matrix Reasoning	0	19	12.83	3.30	0.08 **	0.008
Block Design	4	21	12.45	3.06	0.11 **	<0.001
Visual Puzzles	3	19	12.41	3.14	0.10 **	<0.001
Coding	5	19	12.20	3.01	0.08 *	0.017

* *p* < 0.05, ** *p* < 0.01; Explanation of symbols: Min—minimum score; Max—maximum score; M—mean; SD—standard deviation; Z—result of the Kolmogorov–Smirnov test; *p*—significance. Source: author’s own work.

**Table 3:** Chronic disease and intelligence—Kruskal–Wallis H test and Dunn’s test with the Bonferroni correction.

	Diabetes (n = 88)		Short Stature (n = 38)		Healthy (n = 40)		Post Hoc	H	*p*	ε2R
	M	SD	M	SD	M	SD				
Overall IQ	113.64	12.30	117.97	16.43	113.15	10.72	-	2.20	0.333	-
Verbal IQ	111.16	13.59	112.97	15.67	111.35	9.96	-	0.48	0.788	-
Non-Verbal IQ	113.75	12.89	117.00	16.54	112.48	12.42	-	2.50	0.286	-
Information	10.19	3.14	11.84	3.24	11.58	2.15	1 < 2.3	10.38 **	0.006	0.07
Similarities	12.77	2.55	12.03	3.04	13.05	2.69	-	2.09	0.352	-
Arithmetic	11.43	2.98	11.87	3.44	10.95	2.36	-	2.72	0.257	-
Vocabulary	11.26	2.83	11.42	2.65	11.35	2.24	-	0.28	0.868	-
Comprehension	12.82	2.66	12.84	2.96	11.63	2.50	1.2 > 3	5.81 *	0.050	0.04
Digit Span	10.59	3.08	10.95	2.43	10.10	2.52	-	1.27	0.530	-
Picture Completion	10.23	2.53	11.00	2.76	10.28	2.01	-	2.02	0.365	-
Matrix Reasoning	12.98	3.45	13.00	3.82	12.35	2.28	-	1.66	0.436	-
Block Design	12.09	2.69	13.87	3.59	11.90	2.95	1.3 < 2	9.01 *	0.011	0.05
Visual Puzzles	12.11	3.23	12.47	3.47	13.00	2.55	-	2.39	0.302	-
Coding	12.32	3.20	12.74	2.83	11.45	2.65	-	5.07	0.079	-

* *p* < 0.05, ** *p* < 0.01; Explanation of symbols: M—mean; SD—standard deviation; H—result of the Kruskal–Wallis H test; *p*—significance; ε2R—effect strength coefficient. Source: author’s own work.

**Table 4:** Age according to type of chronic disease and intelligence—Spearman’s rho correlation coefficients.

	Age of Children with Diabetes	Age of Children Suffering from Short Stature	Age of Healthy Children
Overall IQ	−0.167	−0.113	0.144
Verbal IQ	−0.203 *	−0.221	−0.051
Non-Verbal IQ	−0.109	0.020	0.261
Information	−0.093	−0.085	−0.050
Similarities	−0.276 **	−0.282 *	−0.050
Arithmetic	0.150	0.056	−0.040
Vocabulary	−0.365 **	−0.342 *	0.000
Comprehension	−0.085	−0.250	−0.016
Digit Span	0.312 **	0.143	−0.012
Picture Completion	−0.105	−0.178	0.123
Matrix Reasoning	−0.117	0.111	0.048
Block Design	−0.120	0.065	0.126
Visual Puzzles	0.145	0.320 *	0.448 **
Coding	−0.151	−0.023	0.179

* *p* < 0.05, ** *p* < 0.01; Source: author’s own work.

## Data Availability

Data are available upon reasonable request.
